# Commissioning a fast Monte Carlo dose calculation algorithm for lung cancer treatment planning

**DOI:** 10.1120/jacmp.v9i2.2702

**Published:** 2008-04-29

**Authors:** Jeff Craig, Mike Oliver, Adam Gladwish, Matt Mulligan, Jeff Chen, Eugene Wong

**Affiliations:** ^1^ Department of Physics London Regional Cancer Program; ^2^ Departments of Medical Biophysics University of Western Ontario London Ontario Canada; ^3^ Departments of Oncology University of Western Ontario London Ontario Canada; ^4^ Departments of Physics and Astronomy University of Western Ontario London Ontario Canada

**Keywords:** Monte Carlo, commissioning, dose, calculations

## Abstract

A commercial Monte Carlo simulation package, NXEGS 1.12 (NumeriX LLC, New York, NY), was commissioned for photon‐beam dose calculations. The same sets of measured data from 6‐MV and 18‐MV beams were used to commission NXEGS and Pinnacle 6.2b (Philips Medical Systems, Andover, MA). Accuracy and efficiency were compared against the collapsed cone convolution algorithm implemented in Pinnacle 6.2b, together with BEAM simulation (BEAMnrc 2001: National Research Council of Canada, Ottawa, ON). We investigated a number of options in NXEGS: the accuracy of fast Monte Carlo, the re‐implementation of EGS4, post‐processing technique (dose de‐noising algorithm), and dose calculation time. Dose distributions were calculated with NXEGS, Pinnacle, and BEAM in water, lung‐slab, and air‐cylinder phantoms and in a lung patient plan. We compared the dose distributions calculated by NXEGS, Pinnacle, and BEAM. In a selected region of interest (7725 voxels) in the lung phantom, all but 1 voxel had a γ (3% and 3 mm thresholds) of 1 or less for the dose difference between the NXEGS re‐implementation of EGS4 and BEAM, and 99% of the voxels had a γ of 1 or less for the dose difference between NXEGS fast Monte Carlo and BEAM. Fast Monte Carlo with post‐processing was up to 100 times faster than the NXEGS re‐implementation of EGS4, while maintaining ±2% statistical uncertainty. With air inhomogeneities larger than 1 cm, post‐processing preserves the dose perturbations from the air cylinder. When 3 or more beams were used, fast Monte Carlo with post‐processing was comparable to or faster than Pinnacle 6.2b collapsed cone convolution.

PACS numbers: 87.18.Bb, 87.53.Wz

## I. INTRODUCTION

Accurate and fast dose calculation plays an important role in treatment planning for radiotherapy. Various dose calculation algorithms have been used for commercial treatment planning systems, including the pencil beam algorithm,^(^ ^1^ ^–^ ^3^ ^)^ three‐dimensional convolution,^(^ ^4^ ^,^ ^5^ ^)^ collapsed cone convolution (CCC),^(6)^ and Monte Carlo methods.^(^ ^7^ ^–^ ^12^ ^)^ Monte Carlo methods are the most accurate at calculating dose, but they have not been widely used in treatment planning systems (especially for photons) because of slow calculation speed, complex commissioning routines, and lack of resources committed by vendors of treatment planning systems. The CCC method is a good compromise between accuracy and speed, and it is widely used in commercial treatment planning systems. However, differences greater than 5% have been found at the interfaces of materials with different densities, such as at the interface between lung and tissue.^(^ ^13^ ^,^ ^14^ ^)^ A 5% difference is deemed unacceptable, given that an uncertainty of ±2% or better for dose distributions is usually sought to achieve an overall uncertainty of ±5% in delivering the dose to the patient.^(^ ^15^ ^,^ ^16^ ^)^

Monte Carlo simulation consists using well‐established interaction probability distributions to track individual interactions of electrons and photons through a representation of a patient's anatomy. For a Monte Carlo simulation to be accurate, a large number of histories have to be simulated, reducing the statistical uncertainties. For a given number of histories simulated, *N*, the standard deviation of the mean is proportional to $1/N^{1/2}$, and for dose, *D*, within a scoring region, the relative statistical error is proportional to $1/D^{1/2}$.^(17)^ To eliminate all statistical uncertainties from the calculation, an infinite amount of time would have to be devoted to calculating the dose distribution, and so an acceptable level of random uncertainties must be accepted.

Unlike the case with the CCC method, the time required to run a Monte Carlo simulation is independent of the number of beams used; instead, it depends on the number of histories. For a limited number of beams, the CCC method may be able to produce accurate results quickly; however, as the number of beams increases, so does the calculation time. For multiple‐field techniques such as intensity‐modulated radiotherapy (IMRT), intensity‐modulated arc therapy (IMAT),^(18)^ and helical tomotherapy,^(19)^ dose calculation time can be shortened by using Monte Carlo instead of CCC.

With an improving price‐to‐performance ratio for modern computers, fast and accurate Monte Carlo simulations are emerging in modern radiotherapy centers. In the past, Monte Carlo was known to be very accurate, but time‐consuming. NumeriX LLC (New York, NY) has made several approximations and enhancements to their Monte Carlo algorithm—for example, using a multi‐source model instead of simulating the entire head of the linear accelerator, employing variance reduction techniques, re‐implementing the EGS4^(20)^ code into C++ with no change in physics, and post‐processing (de‐noising). This accelerated version of Monte Carlo simulation, called “fast Monte Carlo” by NumeriX, claims to retain the accuracy of previous Monte Carlo implementations (EGS4), but with acceleration of the computation speed.

In the present work, we compared the NumeriX Monte Carlo simulation package, NXEGS 1.12, against the Pinnacle 6.2b CCC algorithm and BEAM 2001 from the National Research Council of Canada (NRC),^(21)^ which we considered to be the “gold standard.” We used BEAM primarily to validate NXEGS dose calculation accuracies and Pinnacle as the reference to compare computation times. The NXEGS package is capable of calculating photon and electron^(22)^ distributions alike; however, the focus in the present work was on photons.

We inspected two calculation algorithms, one being a re‐implementation of EGS4 (EGS4‐NX) and the other being fast Monte Carlo (FMC‐NX). We used water, lung‐slab, and air‐cylinder phantoms and a lung patient plan to evaluate each algorithm. The effect of post‐processing and error estimation in NXEGS for the automated termination of simulation were also investigated. We treated NXEGS as a “black box,” because the exact details of the algorithms and their implementations are not published. In particular, the variance reduction techniques and the post‐processing techniques are proprietary. Several published papers^(^ ^23^ ^–^ ^25^ ^)^ have investigated the efficiency and accuracy of de‐noising algorithms; however, the focus of the present work was the commissioning of the NXEGS photon‐beam package. We therefore investigated post‐processing in conjunction with dose calculation.

## II. METHODS

### A. Commissioning

All three software packages, NXEGS 1.12, Pinnacle 6.2b, and BEAM 2001 were commissioned and validated using measurements from a Varian 2100C/D linear accelerator with both 6‐MV and 18‐MV photon beams.

For NXEGS and Pinnacle, the data required for commissioning consisted of the machine calibration condition, relative dose factors, relative output factors, percent depth dose, and dose profiles. The profiles used to commission the 18‐MV beam consisted of 6 field sizes (5×5 cm, 10×10 cm, 15×15 cm, 20×20 cm, 35×35 cm, and 40×40 cm), and those to commission the 6‐MV beam consisted of 7 field sizes (the earlier 6 sizes, plus 3×3 cm). A tool supplied with NXEGS was used to automatically generate beam parameters for the two photon beams.

### B. Phantoms

Three phantoms, simulating a variety of conditions, were used to test the performance and accuracy of NXEGS (FMC‐NX and EGS4‐NX). The results were compared to CCC and BEAM dose calculations.

#### B.1 Phantom A

A 20×40×20‐cm water phantom (phantom A) with a voxel size of 0.2×0.2×0.2 cm was used to check the output and beam profiles, and to investigate whether NXEGS calculated both the rounded multileaf collimator (MLC) leaves and the tongue‐and‐groove effects. The 6‐MV and 18‐MV beams were used with 4 different setups on this phantom, 1 to collimate the beam to 10×10 cm using only the jaws, and the other 3 with the jaws set to 5×5 cm, but using the MLC leaves to collimate the beam to 3×3 cm, 2×2 cm, and 1×1 cm.

#### B.2 Phantom B

**Figure 1:**
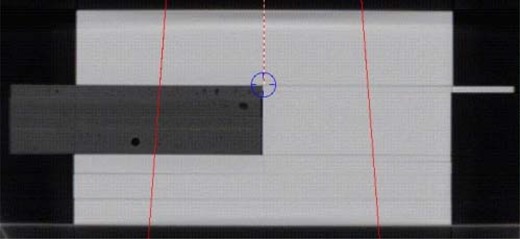
Phantom B, showing the slab of lung‐equivalent material encompassed by Solid Water (Gammex rmi, Middleton, WI) and a 15×15‐cm anterior beam. Dimensions of the phantom are 31×30×17 cm with a resolution of 0.5×0.176×0.176 cm for the dose grid.

A phantom consisting of a set of Solid Water (Gammex rmi, Middleton, WI) and lung equivalent material blocks [phantom B (Fig. 1)] underwent computed tomography (CT) imaging to test the dose calculation with a large inhomogeneity for each algorithm. The phantom itself measured approximately 31×30×17 cm, with a lung slab of 20×5.5×20 cm. We used a common dose grid of 35×45×45 cm with a voxel size of 0.5×0.176×0.176 cm for all algorithms. An anterior beam (6 MV or 18 MV), a source‐to‐surface distance (SSD) of 104 cm, and an isocenter located 2.5 cm above the center of the phantom were used to calculate dose distributions. Fields were sized to 15×15 cm, 10×10 cm, 5×5 cm, 3×3 cm, and 10×10 cm, with the MLC set to 5×5 cm.

#### B.3 Phantom C

The final phantom (phantom C) was created manually in TheraPlan Plus (Nucletron, Veenendaal, Netherlands), version 3.8, and exported as a CT image to test how FMC‐NX, EGS4‐NX, and CCC handled small inhomogeneities. Phantom C consisted of water (15×15×20 cm) with an air cylinder of diameter 1, 2, or 3 cm placed with its center at an equal distance from the top and the bottom of the phantom. The dose grid was 15×24.9×24.9 cm with a voxel size of 0.3×0.3×0.3 cm for all algorithms. An anterior beam (18 MV, 5×5 cm) was used for each case with an isocenter set to the middle of the air cylinder.

#### B.4 Lung Patient

**Figure 2:**
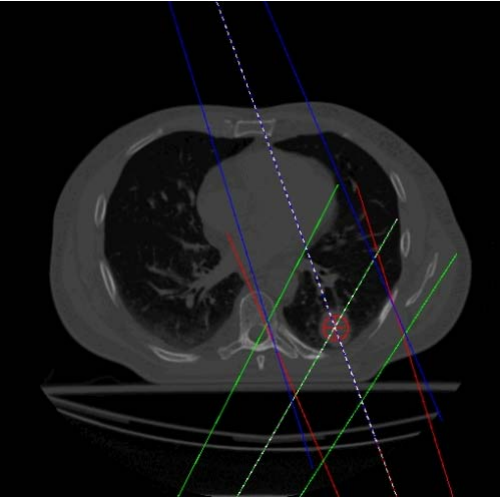
Lung patient with tumor in posterior region of the left lung (dose grid resolution of 0.25×0.25×0.25 cm).

A clinical lung cancer case was used to compare FMC‐NX with post‐processing and EGS4‐NX against CCC (Fig. 2). The plan consisted of three 18‐MV fields conforming to the planning target volume with gantry angles 160, 210, and 340 degrees, with the isocenter set to the center of the planning target volume as shown in Fig. 2. The CT image was 18×40×40 cm, with a voxel size of 0.25×0.25×0.25 cm for the dose grid.

### B. Comparison

For comparison purposes, all doses were converted to centigrays per monitor unit (MU) by specifying the calibrated dose rate (0.903 cGy/MU for 18 MV; 0.848 cGy/MU for 6 MV) under the calibration conditions. For the 6‐MV and 18‐MV beams alike, the calibration conditions consisted of a 10×10‐cm field, a 90‐cm SSD, and a 10‐cm depth (which is equivalent to 1 cGy/MU with a 10×10‐cm field and 95‐cm SSD at a depth of 5 cm). Unless otherwise specified, all dose distributions were calculated so that the dose had a relative standard deviation of ±2% at isocenter.

The Computational Environment for Radiotherapy Research^(26)^ was used to display and compare the various dose distributions from the various algorithms.

The gamma index^(27)^ was calculated for every phantom on the central slice of the dose distribution, taken from a section 4 cm below the top of the phantom and 9 cm either side of the central axis. This technique was selected to avoid any bias in the gamma values resulting from electron contamination or from the low dose values outside the range of the beam. The criteria used for the gamma‐index calculations were 3% dose and 3 mm distance to agreement.

## III. RESULTS

### A. Commissioning

On a 3‐GHz Pentium 4 processor, it took 29 CPU hours in two steps for the automated commissioning tools in NXEGS to commission and verify the results of the 18‐MV beam against the input data. The first step, to model the beam, took 16 hours with 100 million histories using Monte Carlo and analytic methods. The second step, running a simulation of the input data, took 13 hours, and the number of histories used for each field varied from 6.4 million for the 5×5‐cm field up to 40 million for the 40×40‐cm field.

**Figure 3:**
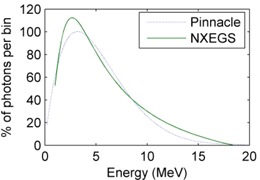
The 18‐MV beam spectra produced after commissioning NXEGS (NumeriX LLC, New York, NY) and Pinnacle 6.2b, with both spectra normalized to the area under the curve.

The NXEGS and Pinnacle 6.2b calculations both produced accuracies in water that, when compared with the experimental data, were similar. The results from the NXEGS calculations were within ±1% as compared with the measured input data. Similar spectra^(28)^ were also produced by NXEGS and Pinnacle 6.2b during commissioning, when normalized to the area under the curve (Fig. 3).

### B. Dose comparison

#### B.1 Phantom A

**Figure 4:**
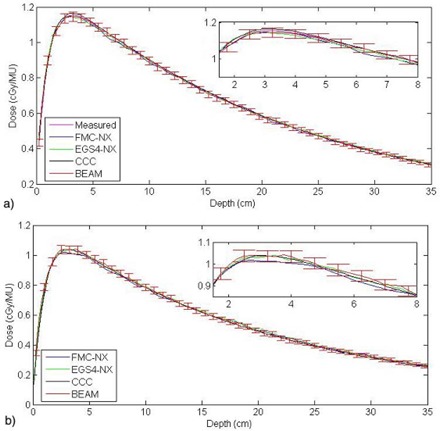
(a) Depth dose for a 10×10‐cm field with a source‐to‐surface distance of 90 cm using an 18‐MV beam on phantom A, with a difference of 0.8% at $D_{\max}$ between FMC‐NX [fast Monte Carlo code, NumeriX LLC (New York, NY) implementation] and the measured data. (b) Depth dose for a 3×3 cm multileaf collimator field with the jaws set to 5×5 cm in phantom A, using a beam energy of 18 MV with a source‐to‐surface distance of 90 cm. MU=monitor unit; EGS4‐NX=EGS4 Monte Carlo code,NumeriX LLC (New York,NY) implementation; CCC=collapsed cone convolution,Pinnacle implementation; BEAM=BEAMnrc code,National Research Council of Canada implementation.

For the 18‐MV beam, the depth doses from FMC‐NX, EGS4‐NX, BEAM, and CCC agreed within ±2% of the measured data [Fig. 4(a)]. The dose calculated by CCC was slightly higher (by 1.2%) as compared with the measured data at the depth of maximum dose ($D_{\max}$); the doses calculated by FMC‐NX and BEAM were slightly lower (by 1%) than the measured dose. All algorithms begin to converge to the measured data at deeper depth. The same trend is seen with the 6‐MV data, except at $D_{\max}$, where the difference is about 2% for FMC‐NX as compared with the measured data, but the results converge to within 1.5%−1% difference at deeper depths.

Neither NXEGS nor Pinnacle 6.2b was commissioned with a field size smaller than 5×5 cm for the 18‐MV beam and 3×3 cm for the 6‐MV beam; however, we investigated the model accuracies with field sizes down to 1×1 cm in water. A point dose measurement, taken with a 0.12 cm^3^ Scanditronix ion chamber (Scanditronix–Wellhofer, Nuremburg, Germany) for a 3×3‐cm field at a depth of 10 cm, yielded 0.785 cGy/MU. Comparatively, CCC showed a difference of 0.3% (0.004 cGy/MU); BEAM, 1.3% (0.017 cGy/MU); FMC‐NX, 1.6% (0.021 cGy/MU); and EGS4‐NX, 2.3% (0.03 cGy/MU). The NXEGS and Pinnacle software both used beam models, and the commissioning data was supplied only for field sizes down to 5×5 cm, and so both had to extrapolate to 3×3 cm. When all distributions are normalized to 0.785 cGy/MU at a depth of 10 cm, we observe that, at a depth of about 2−3 cm, the difference in dose between CCC and FMC‐NX is about 0.02 cGy/MU [Fig. 4(b)]. Differences of a similar magnitude were observed for field sizes smaller than 3×3 cm.

#### B.2 Phantom B

**Figure 5:**
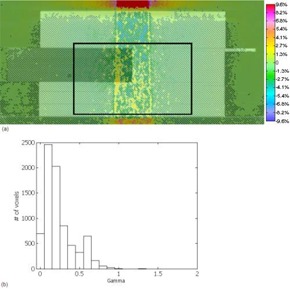
(a) Subtraction of BEAM (BEAMnrc code, National Research Council of Canada implementation) dose from EGS4‐NX [EGS4 Monte Carlo code, NumeriX LLC (New York, NY) implementation] dose. Dose scale from −9.6% (−0.07% cGy per monitor unit) to +9.6% (+0.07% cGy per monitor unit). Dose was calculated using a 5×5 cm 18‐MV beam with a dose grid of 0.5×0.176×0.176 cm. Black box indicates the area used for gamma calculations. (b) Gamma values taken from the boxed region shown in (a), with 3% and 3 mm parameters and 7724 of 7725 voxels having a γ of 1 or less.

For both 18‐MV and 6‐MV beams, doses with EGS4‐NX were about 2% lower than were those with BEAM [see the 18‐MV beam results in Fig. 5(a)]. Fig. 5(b) shows the gamma histogram of the dose difference within the region indicated by the black box in Fig. 5(a). Within this selected region of interest (7725 voxels), all but 1 voxel had a γ (3% and 3 mm thresholds) of 1 or less. Any major differences in the dose distributions would be attributed to the NXEGS beam modeling.

**Figure 6:**
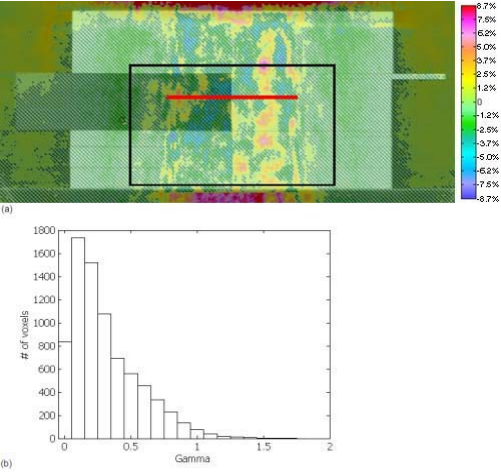
(a) Subtraction of BEAM (BEAMnrc code, National Research Council of Canada implementation) dose from FMC‐NX [fast Monte Carlo code, NumeriX LLC (New York, NY) implementation] dose. Dose calculated using an 18‐MV beam set to a 10×10 cm field. Displayed dose ranging from −8.7% (−0.07% cGy per monitor unit) to +8.7% (+0.07% cGy per monitor unit). The red line indicates where the profile for Fig. 8 was taken. (b) Gamma values taken from the boxed region shown in (a), with 3% and 3 mm parameters and 7640 of 7725 voxels having a γ of 1 or less.

Fig. 6(a) shows the dose difference between FMC‐NX with post‐processing (de‐noising) and BEAM, and Fig. 6(b) shows the corresponding gamma index histogram in the region indicated by the black box in Fig. 6(a). Of all the voxels, 99% had a γ (3% and 3 mm thresholds) of 1 or less for the dose difference. These results indicate that FMC‐NX is not as accurate as EGS4‐NX, but that, overall, most of the dose distribution falls within ±0.03 cGy/MU of BEAM. With FMC‐NX, not only can the beam modeling cause differences in the dose distribution, but the EGS4 algorithm has also been modified to reduce the calculation time and the number of histories. Another possibility for introducing uncertainty into the dose distributions is the use of fewer histories in conjunction with post‐processing to reduce statistical variations.

**Figure 7:**
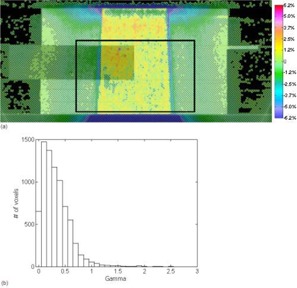
(a) Subtraction of BEAM (BEAMnrc code, National Research Council of Canada implementation) dose from CCC (collapsed cone convolution, Pinnacle implementation) dose, using an 18‐MV beam with a 10×10 cm field. Dose difference ranges from −6.2% (−0.05% cGy per monitor unit) to +6.2% (+0.05% cGy per monitor unit). Black box indicates the area used for gamma calculations. (b) Gamma values taken from the boxed region shown in (a), with 3% and 3 mm parameters.

**Figure 8:**
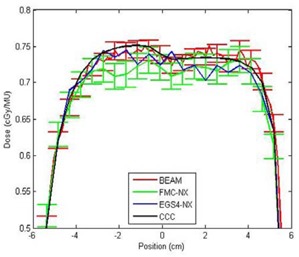
Crossline profiles taken at a depth of 8.5 cm on phantom B for FMC‐NX [fast Monte Carlo code, NumeriX LLC (New York, NY) implementation], EGS4‐NX [EGS4 Monte Carlo code, NumeriX LLC (New York, NY) implementation], BEAM (BEAMnrc code, National Research Council of Canada implementation), CCC (collapsed cone convolution, Pinnacle implementation), with a field size of 10×10 cm and 18‐MV energy. MU = monitor units.

To provide a metric to compare the differences between FMC‐NX and BEAM, we show in Fig. 7(a) the difference in dose distribution of a 10×10‐cm, 18‐MV beam calculated by CCC in Pinnacle and by BEAM. Fig. 7(b) shows the corresponding gamma index histogram. This set of figures is meant to be compared with the FMC‐NX results in Fig. 6(a,c). Fig. 8 shows the dose profile comparisons at a depth of 8.5 cm for the beams calculated in Figs. 6(a) and 7(a), comparing the doses calculated by FMC‐NX, EGS4‐NX, BEAM, and CCC.

**Figure 9:**
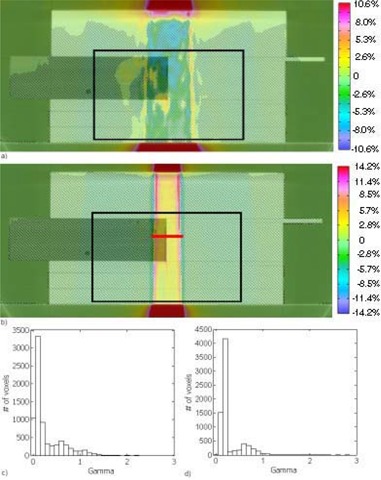
(a) Subtraction of CCC (collapsed cone convolution, Pinnacle implementation) from FMC‐NX [fast Monte Carlo code, NumeriX LLC (New York, NY) implementation], with an 18‐MV beam and field set to 5×5 cm. Dose values range from −10.6% (−0.08% cGy per monitor unit) to +10.6% (−0.08% cGy per monitor unit). (b) The 6‐MV 3×3 cm beam shows a wider penumbra for collapsed cone. Subtraction of CCC from FMC‐NX, with dose ranging from −14.2% (−0.1% cGy per monitor unit) to +14.2% (+0.1% cGy per monitor unit). The red line indicates the location from which the profile for Fig. 11 was taken. (c,d) Gamma values taken from the regions shown in (a) and (b) respectively, with 3% and 3 mm parameters.

**Figure 10:**
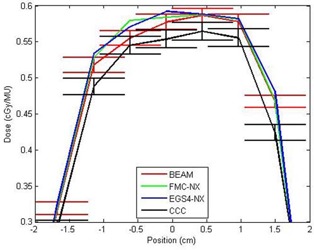
Crossline profiles taken at a depth of 8.5 cm on phantom B for FMC‐NX [fast Monte Carlo code, NumeriX LLC (New York, NY) implementation], EGS4‐NX [EGS4 Monte Carlo code, NumeriX LLC (New York, NY) implementation], BEAM (BEAMnrc code, National Research Council of Canada implementation), CCC (collapsed cone convolution, Pinnacle implementation), with a field size of 3×3 cm and 6‐MV energy.

Figs. 9(a) and 9(c) provide a direct comparison between CCC and FMC‐NX for an 18‐MV beam with a field size of 5×5 cm. Figs. 9(b) and 9(d) provide a direct comparison between CCC and FMC‐NX for a 6‐MV beam with a field size of 3×3 cm. Fig. 10 shows line profiles at depth of 8.5 cm, comparing the doses calculated by FMC‐NX, EGS4‐NX, CCC, and BEAM.

For the 18‐MV data, the FMC‐NX algorithm calculates the dose in the penumbra better than does CCC: the CCC dose is shifted by 3 mm toward the central axis as compared with the BEAM dose [Fig. 7(a)]. Conversely, the noise is absent in CCC, but present in all Monte Carlo dose calculations. Furthermore, we found that the electron contamination in the buildup region for field sizes smaller than 5×5 cm is modeled better by FMC‐NX than by CCC in Pinnacle 6.2b.

In the 6‐MV data, CCC had a wider penumbra for all field sizes (by about 3 mm on either side), except in the lung portion of the phantom, where the penumbra agreed with that calculated by BEAM and NXEGS (Fig. 10).

#### B.3 Phantom C

**Figure 11:**
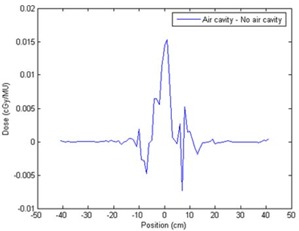
Dose profile of phantom C taken 4 cm below the 1‐cm air cylinder. Dose simulated using FMC‐NX [fast Monte Carlo code, NumeriX LLC (New York, NY) implementation] with post‐processing to a standard deviation of ±0.2%. MU=monitor units monitor units.

To detect whether post‐processing washes out small inhomogeneities, we used a voxel located 4 cm below each air cylinder to compare dose values (Table 1). Calculations by FMC‐NX were completed with and without post‐processing, and CCC calculations were also performed. To determine what the results would look like if post‐processing indeed washed out the inhomogeneities, calculations with the same phantom were repeated, except that the air cylinders were filled with water, creating a homogenous medium. Dose values agreed well with each other to within ±2%, which would indicate that with post‐processing, inhomogeneities as small as 1 cm in diameter will not be washed out. Two simulations were also performed using 10 million histories for FMC‐NX, with post‐processing for the phantom with and without the 1‐cm air cylinder. Both calculations fell within a standard deviation of ±0.2%. A profile (Fig. 11) was taken of the dose difference 4 cm below the 1‐cm air cylinder.

#### B.4 Lung Patient

Three 18‐MV beams, with gantry angles of 160, 210, and 340 degrees, were set to conform to the planning target volume. Table 2 reports the dose values at isocenter. Because previous results have shown that EGS4‐NX simulation is comparable to BEAM, we did not plan the patient with BEAM. The couch was not included in the plans.

**Table 1:** Comparing a dose point for dose distributions prepared with and without post‐processing,^a^ to 2 standard deviations

	*Phantom C with air pocket*		*Phantom C without air pocket*			
	1 cm	2 cm	3 cm	1 cm	2 cm	3 cm
FMC‐NX^b^ with post‐processing (cGy/MU)	0.7262	0.7377	0.7282	0.7230	0.7069	0.6814
FMC‐NX^b^ without post‐processing (cGy/MU)	0.7203	0.7337	0.7395	0.7169	0.7146	0.6735
CCC^c^ (cGy/MU)	0.7307	0.7362	0.7322	0.7163	0.7073	0.687

a In each case, the dose point is 4 cm below the bottom of the air cylinder. Dose distributions were all calculated on the same phantom; for “without air pocket” measurements, the air cylinder was filled with water, making a homogenous medium.

b Fast Monte Carlo code, NumeriX LLC (New York, NY) implementation.

c Collapsed cone convolution, Pinnacle implementation.

MU = monitor unit.

**Figure 12:**
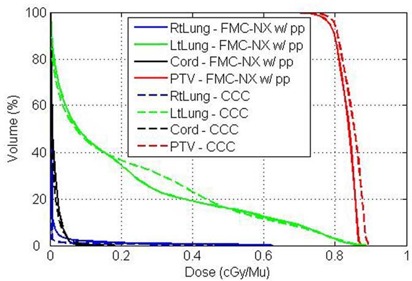
Dose–volume histogram of lung patient, comparing FMC‐NX [fast Monte Carlo code, NumeriX LLC (New York, NY) implementation] with post‐processing (solid lines) and CCC [collapsed cone convolution, Pinnacle implementation (dotted lines)] using three 18‐MV beams.

When the dose calculation algorithms are compared using a dose–volume histogram, a small discrepancy can be observed between CCC and FMC‐NX with post‐processing (Fig. 12; for readability, FMC‐NX without post‐processing and EGS4‐NX have been omitted).

**Figure 13:**
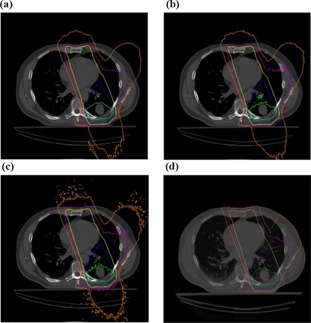
(a) FMC‐NX [fast Monte Carlo code, NumeriX LLC (New York, NY) implementation] with post‐processing. (b) FMC‐NX without post‐processing. (c) EGS4‐NX [EGS4 Monte Carlo code, NumeriX LLC (New York, NY) implementation]. (d) CCC (collapsed cone convolution, Pinnacle implementation). Isodose lines are at 105% (red), 95% (green), 80% (blue), 50% (yellow), 25% (purple), and 10% (orange) of the dose at isocenter.

When the plan is normalized to the isocenter dose, all algorithms produce similar isodose distributions (Fig. 13). Table 3 reports the differences in calculation times.

### C. Time

Looking at the calculation time for phantom B, the CCC algorithm is seen to be the fastest, followed by FMC‐NX using post‐processing, EGS4‐NX, and BEAM (Table 4). All calculations were taken to ±2% error at isocenter. The CCC computation time increases linearly with added beams, but the Monte Carlo calculation times depend only on the number of histories simulated. The use of post‐processing adds about 1 minute for every $10^{6}$ dose voxels being computed, but the reduction in the number of histories required to reach the same accuracy produces a faster calculation time.

**Table 2:** Dose at the isocenter for each beam simulated separately and for all beams added together

*NumeriX*				*Pinnacle 6.2b*
	*FMC‐NX*		*EGS4‐NX*	*CCC*
	*With post‐processing*	*Without post‐processing*		
160 degrees (cGy/MU)	0.94425	0.94090	0.95006	0.97774
210 degrees (cGy/MU)	0.94475	0.95426	0.96963	0.96341
340 degrees (cGy/MU)	0.69169	0.68467	0.69266	0.69919
Plan with equal MU weighting (cGy/MU)	0.86023	0.85994	0.87078	0.88011

FMC‐NX=fast Monte Carlo code,NumeriX LLC (New York,NY) implementation; EGS4‐NX=EGS4 Monte Carlo code,NumeriX LLC (New York,NY) implementation; CCC=collapsed cone convolution,Pinnacle implementation; MU=monitor units.

**Table 3:** Time and number of histories used to calculate dose on a 160×by160×72‐voxel grid on the lung patient to ±2% error at isocenter^a^

*NumeriX*				*Pinnacle 6.2b*
	*FMC‐NX*		*EGS4‐NX*	*CCC*
	*With post‐processing*	*Without post‐processing*		
Time (min)	3.15	27	183	6.25
Histories (*n*)	300 000	7 000 000	70 000 000	N/A

a Time is for a total of 3 beams; however, because NXEGS (NumeriX LLC, New York, NY) required the same number of histories to achieve ±2% error at the isocenter, the time for 3 beams or for 1 beam are the same. Pinnacle time has to be divided by 3 to determine the time needed to calculate dose for 1 beam.

FMC‐NX=fast Monte Carlo code,NumeriX LLC (New York,NY) implementation; EGS4−NX=EGS4 Monte Carlo code,NumeriX LLC (New York,NY) implementation; CCC=collapsed cone convolution,Pinnacle implementation.

**Table 4:** Times and number of histories used by each dose calculation algorithm for phantom B with a 256×256×64‐voxel dose grid, all calculations done to ±2% error at the isocenter

		*FMC‐NX*		*EGS4‐NX*		*BEAM*		*CCC*	
*Jaw (cm^2^)*	*Energy (MV)*	*Time (min)*	*Histories* $\left(\times 10^{5}\right)$	*Time (min)*	*Histories* $\left(\times 10^{5}\right)$	*Time (min)*	*Histories* $\left(\times 10^{5}\right)$	*Time (min)*	*Histories* $\left(\times 10^{5}\right)$
15×15	18	10.40	10	928.15	2 000	9960	3 500	3.8	N/A
	6	7.15	5	707.96	3 000	6918	3 500	3.8	N/A
10×10	18	6.86	4	462.46	1 000	8820	3 000	3.6	N/A
	6	5.65	2	534.86	1 500	6432	3 000	3.6	N/A
10×10w/MLC	18	5.68	7	133.38	1 000	8124	3 000	3.6	N/A
	6	5.50	6	158.30	1 500	6330	3 000	3.6	N/A
5×5	18	5.68	2	138.03	300	5280	1 000	3.2	N/A
	6	5.15	1	180.46	500	3642	1 000	3.3	N/A
3×3	18	5.50	1.7	22.73	50	3120	500	3.1	N/A
	6	5.15	1	104.55	300	2760	500	3.2	N/A

a All NXEGS (NumeriX LLC, New York, NY) simulations were run on a Pentium 4 3 GHz processor; BEAM (BEAMnrc code: National Research Council of Canada, Ottawa, ON) calculations were run on a 1.8 GHz Xeon processor; and Pinnacle calculations were run on a Sun Blade system.

FMC‐NX=fast Monte Carlo code,NumeriX LLC implementation; EGS4−NX=EGS4 Monte Carlo code,NumeriX LLC implementation; BEAM=BEAMnrc code,National Research Council of Canada implementation; CCC=collapsed cone convolution,Pinnacle implementation; N/A=not available; w/MLC=with multileaf collimator.

## IV. DISCUSSION

Pinnacle 6.2b did not model rounded MLC leaves, but that option is available in a newer release of Pinnacle (version 7.6c). In NXEGS, the rounded MLC can be modeled with discretized rectangular blocks. Similarly, tongue‐and‐groove can be modeled using blocks of varying size.

We used BEAMnrc 2001 in the present work. Afterwards, BEAMnrc 2006 was released, with increases in calculation speed of approximately 6.4 times (6 MV beams) and 3.5 times (18 MV beams) that of BEAMnrc 2001. The disadvantage of using NXEGS is that it lacks the history and publication record of BEAM or EGS4. The advantages of NXEGS include ease of commissioning (similar to that of Pinnacle) and, more importantly, its focus on ease of software integration, with its documented application programming interface.

When a dose distribution falls within a region that has only a few small, non‐critical inhomogeneities, then the use of post‐processing with a smaller number of histories will speed up the dose calculation significantly with minimum effect on the accuracy in the calculated dose distribution. However, because of a lack of information on how post‐processing works, and because in the present work we have investigated the effects of inhomogeneities only down to 1 cm, it is advisable that, for areas with small critical structures (such as in the nasosinus), FMC‐NX be used with an increased number of histories and without post‐processing to allow for greater accuracy at the expense of an increase in calculation time.

## V. CONCLUSIONS

The NXEGS software was compared with two dose calculation algorithms, the Pinnacle 6.2b CCC, and the NRC BEAM. We commissioned NXEGS and Pinnacle 6.2b to use 6‐MV and 18‐MV beams. The FMC‐NX algorithm with post‐processing accurately predicted dose spread resulting from electron transport in lung. Using only a 3‐source model, NXEGS predicted accurate dose distributions for most situations. Post‐processing is applied after the dose distribution has been calculated; it takes about 1 minute per 10^6^ voxels. For an accuracy of ±2% in the target volume, dose calculation time for FMC‐NX with post‐processing is comparable to that for CCC when a treatment plan consists of 3 or more beams. Because Monte Carlo calculation time is based on the number of histories and not on the number of beams, the more beams added, the faster it will be as compared with other systems, making it desirable for IMRT, IMAT, or tomotherapy. The FMC‐NX algorithm with post‐processing lends itself to treatment planning with a good accuracy‐to‐speed ratio.

## ACKNOWLEDGMENTS

Our research was supported in part by the Canadian Cancer Society. The authors also thank Dr. Stewart Gaede for the clinical case and acknowledge the use of a complimentary academic license for NXEGS 1.12 provided by NX Medical Software LLC. We have no commercial interest in NXEGS, and we declare no conflict of interest with NumeriX.
